# Proteomic profiling of milk small extracellular vesicles from bovine leukemia virus-infected cattle

**DOI:** 10.1038/s41598-021-82598-2

**Published:** 2021-02-03

**Authors:** Md. Matiur Rahman, Shigeo Takashima, Yuji O. Kamatari, Yassien Badr, Yuko Kitamura, Kaori Shimizu, Ayaka Okada, Yasuo Inoshima

**Affiliations:** 1 The United Graduate School of Veterinary Sciences, Gifu University, 1-1 Yanagido, Gifu, Gifu 501-1193 Japan; 2 Laboratory of Food and Environmental Hygiene, Cooperative Department of Veterinary Medicine, Gifu University, 1-1 Yanagido, Gifu, Gifu 501-1193 Japan; 3 Department of Medicine, Faculty of Veterinary, Animal and Biomedical Sciences, Sylhet Agricultural University, Sylhet, 3100 Bangladesh; 4 Division of Genomics Research, Life Science Research Center, Gifu University, 1-1 Yanagido, Gifu, Gifu 501-1193 Japan; 5 Division of Instrumental Analysis, Life Science Research Center, Gifu University, 1-1 Yanagido, Gifu, Gifu 501-1193 Japan; 6 Department of Animal Medicine (Branch of Infectious Diseases), Faculty of Veterinary Medicine, Damanhour University, El-Beheira, Egypt; 7 Gifu Prefectural Chuo Livestock Hygiene Service Center, 1-1 Yanagido, Gifu, Gifu 501-1112 Japan; 8 Education and Research Center for Food Animal Health, Gifu University (GeFAH), 1-1 Yanagido, Gifu, Gifu 501-1193 Japan; 9 Joint Graduate School of Veterinary Sciences, Gifu University, 1-1 Yanagido, Gifu, Gifu 501-1193 Japan

**Keywords:** Computational biology and bioinformatics, Microbiology, Diseases

## Abstract

Milk small extracellular vesicles (sEV) contain proteins that provide potential information of host physiology and immunology. Bovine leukemia virus (BLV) is an oncogenic virus that causes progressive B-cell lymphosarcoma in cattle. In this study, we aimed to explore the proteomic profile of milk sEV from BLV-infected cattle compared with those from uninfected cattle. Milk sEV were isolated from three BLV-infected and three uninfected cattle. Proteomic analysis was performed by using a comprehensive nanoLC-MS/MS method. Furthermore, gene ontology (GO) annotation and Kyoto Encyclopedia of Genes and Genomes (KEGG) pathways were used to evaluate the candidates for uniquely or differentially expressed proteins in milk sEV from BLV-infected cattle. Proteomic analysis revealed a total of 1330 common proteins in milk sEV among BLV-infected cattle, whereas 118 proteins were uniquely expressed compared with those from uninfected cattle. Twenty-six proteins in milk sEV were differentially expressed proteins more than two-fold significant difference (*p* < 0.05) in BLV-infected cattle. GO and KEGG analyses indicated that the candidates for uniquely or differentially expressed proteins in milk sEV had been involved in diverse biological activities including metabolic processes, cellular processes, respond to stimulus, binding, catalytic activities, cancer pathways, focal adhesion, and so on. Taken together, the present findings provided a novel insight into the proteomes of milk sEV from BLV-infected cattle.

## Introduction

Extracellular vesicles (EV) are membranous particles, secreted by a wide variety of cells found in all biological fluids in humans and animals such as blood, amniotic fluid, ascitic fluid, urine, saliva, tears, and milk^1^. There is still some variability in the different classes of EV that included exosomes, ectosomes or shedding microvesicles, apoptotic bodies, and other EV subsets according to their size, biogenesis, and releasing pathway^2^. Among all classes of EV, one of the EV is classified as small EV (sEV), so called exosomes (30–150 nm in diameter)^1^. The International Society for Extracellular Vesicles (ISEV) has been established to accelerate research activities including all classes of EV. Recently, ISEV suggested that the term “sEV” should be used instead of “exosomes” in the Minimal Information for Studies of Extracellular Vesicles guidelines 2018 (MISEV2018)^3^.

Milk is a diverse source of sEV that contains proteins, microRNAs (miRNAs), mRNAs, DNA, and lipids that play an important role in many biological activities including cell growth, development, immune modulation and regulation^4,5^. For example, sEV derived from beneficial bacteria in human breast milk has been involved in the transfer of gut microbiota from mother to infant^6^. More recent study described that bovine milk sEV also contained proteins, miRNAs, mRNAs, DNA, and lipids that were considered to transport biologically active cargos from donor to recipient cells for exchanging genetic information^7^. Over the last two decades, proteomic analysis has been widely used to detect changes of proteins in milk sEV in relation with many physiological information^8^. A recent study reported that milk sEV contained distinctive proteins that provided the potential information of mammary physiology of cattle^9^. Moreover, the proteomic analysis of milk sEV provided pathological information of disease of cattle. For example, a previous study reported that miRNAs such as bta-miR-142-5p and bta-miR-223 had been up-regulated in milk sEV from *Staphylococcus aureus*-infection, considered as a potential biomarker for monitoring of physiological and pathological status in cattle^10^.

Bovine leukemia virus (BLV) is one of the tumorigenic virus that causes enzootic bovine leukosis (EBL) characterised by B-cell lymphosarcoma and is present worldwide, including Japan^11^. A nationwide survey in Japan reported that approximately 40.9% of dairy cattle and 28.7% of beef cattle had antibodies against BLV^12^. Among cattle infected with BLV, 2–5% of cattle develop clinical signs of B-cell lymphosarcoma and 20–30% of cattle progress to persistent lymphocytosis (PL). Approximately 70% of BLV-infected cattle do not show any clinical signs and remain sub-clinically infected for life^13^. To date, evidence has indicated that host genetic factors may play a vital role in the stages of BLV infection: from early infection to developing of PL to lymphoma^14^. Very recently, our study revealed that BLV infection caused profound effects on host cellular activity resulting in changes of encapsulated mRNA in milk sEV obtained from BLV-infected cattle^15^. We hypothesised that there would be a probable change in encapsulating proteins in milk sEV also during BLV infection in cattle. However, to date, no study has reported on the proteomic analysis of milk sEV from BLV-infected cattle.

In this study, a comprehensive proteomic analysis in milk sEV was performed to identify the changes in protein levels in BLV-infected cattle. The proteomic analysis revealed a large number of proteins along with candidates for uniquely or differentially expressed proteins in milk sEV from BLV-infected cattle. Further, gene ontology (GO) annotation and Kyoto Encyclopaedia of Genes and Genomes (KEGG) pathways provided a new perspective to understand and unveil the pathological roles of milk sEV during BLV infection in cattle. Our study suggested that the candidates for uniquely or differentially expressed proteins in milk sEV could be the land mark for future investigations of clinical stages of BLV infection and it’s pathogenesis in cattle by using of milk sEV.

## Results

### Clinical status of cattle

The clinical status including BLV infection, hematology, and serum chemistry parameters of cattle were assessed and shown in Table 1. BLV provirus and BLV antibody was checked either by nested polymerase chain reaction (nested PCR) and enzyme-linked immunosorbent assay (ELISA) for the confirmation of BLV infection in cattle. Three BLV-infected cattle had high proviral load (HPL) > 30,000/10^5^ white blood cells (WBCs) DNA along with high lactate dehydrogenase isozymes 2 and 3 (LDH2 + 3) percentage > 30%. The high WBCs and lymphocyte counts along with age were indicating either ‘Suspect’ or ‘Lymphocytic’ according to the European Community's (EC) key parameter^16^.

**Table 1:** Clinical status of BLV-infected and uninfected cattle.

Cattle no.^※1^	Age^2^ (month)	ELISA^3^ (antibody)	Nested PCR	Proviral load^4^	WBC^5^ (/µl)	Lymphocyte (/µl)	Key of EC^6^	LDH^7^					
								1	2	3	2 + 3	4	5
BLV-infected cattle													
B1	84	+	+	32,023	17,000	10,931	+	47.7	25.1	16.5	41.6	6.7	4.0
B2	72	+	+	33,480	9900	6752	±	54.5	15.6	15.8	31.4	7.7	6.4
B3	96	+	+	36,859	8900	6070	±	58.4	18.8	13.5	32.1	6.3	3.2
Uninfected cattle													
U1	108	−	−	NT	NT	NT	NT	NT	NT	NT	NT	NT	NT
U2	48	−	−	NT	NT	NT	NT	NT	NT	NT	NT	NT	NT
U3	84	−	−	NT	NT	NT	NT	NT	NT	NT	NT	NT	NT

+ , positive; -, negative; ± , suspect; NT, not tested; ^1^no., number; ^2^Age at the time of blood sampling; ^3^ELISA, anti-BLV antibody enzyme-linked immunosorbent assay; ^4^copies/10^5^ WBCs DNA; ^5^WBCs, white blood cells; ^6^Key of EC, leucosis-key of the European Community; ^7^ LDH, lactate dehydrogenase.

### Protein concentration of milk sEV

Protein concentration of milk sEV from BLV-infected and uninfected cattle were 9.95 mg/mL and 9.92 mg/mL, respectively (*p* > 0.05) (Supplementary Fig. 1).

**Figure 1:**
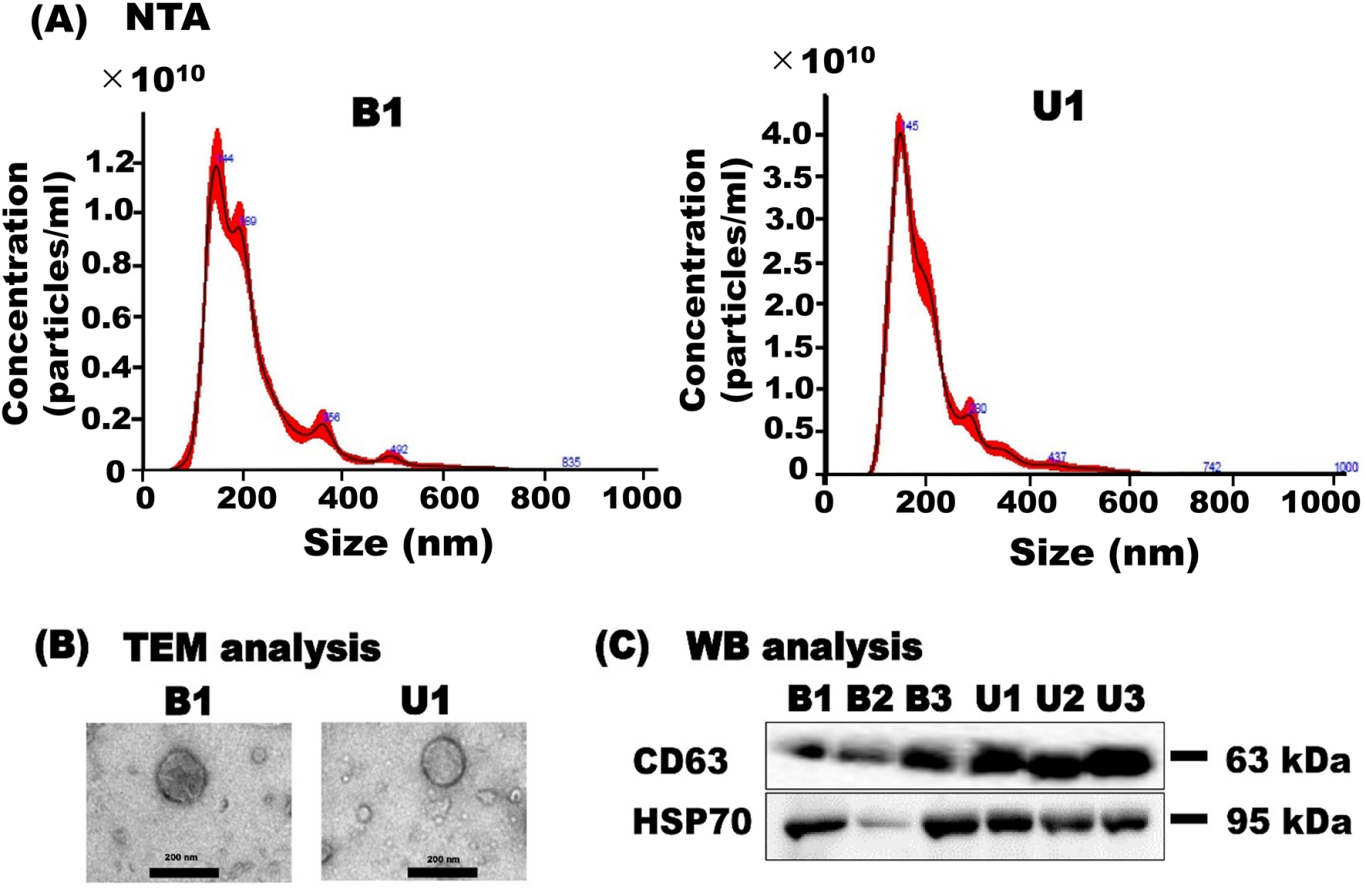
Milk sEV characterization. The peak (mode) of particle size distribution of the milk sEV from BLV-infected and uninfected cattle were showed by NTA. Representative data from B1 and U1 cattle were shown (**A**). Similar spherical morphology of milk sEV between BLV-infected and uninfected cattle were observed by TEM analysis. Scale bar shows 200 nm (**B**). Milk sEV were successfully detected by WB analysis using antibodies against sEV-surface-marker protein CD63 and internal protein HSP70 (C).

### Detection of BLV genomic RNA and BLV proteins in milk sEV

To select BLV genomic RNA and BLV protein free milk sEV from BLV-infected cattle, reverse transcription-nested PCR and western blot (WB) analyses were performed. The results indicated that both BLV genomic RNA and BLV protein were not detected in milk sEV from BLV-infected cattle (Supplementary Fig. 2A and 1B).

**Figure 2:**
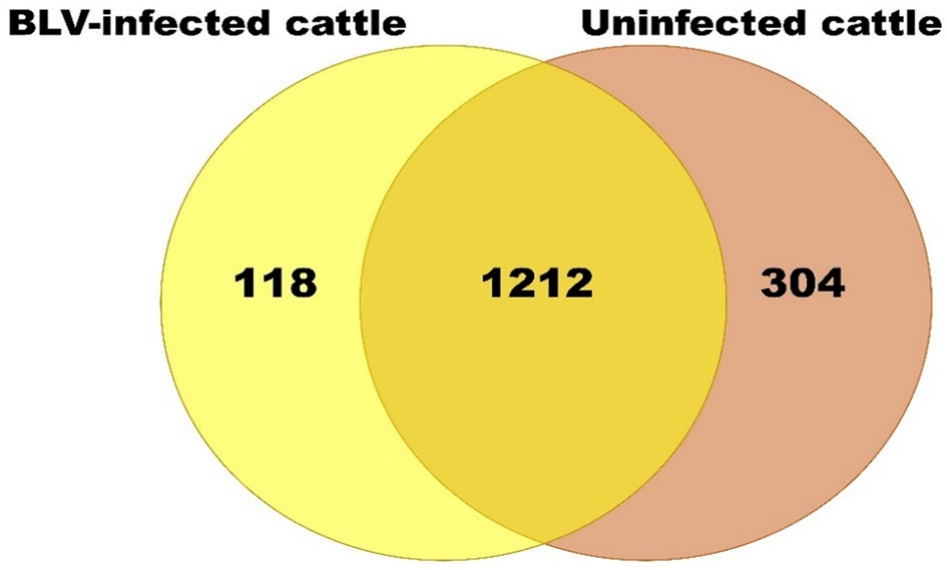
Comparative Venn diagram of milk sEV proteins between BLV-infected and uninfected cattle were shown.

### Characterization of milk sEV

For the characterization of isolated milk sEV from BLV-infected and uninfected cattle biophysically, nanoparticle tracking analysis (NTA) and transmission electron microscopy (TEM) analysis were performed. NTA showed that the peak (mode) for particle size distribution of milk sEV from BLV-infected and uninfected cattle were 145.6 nm and 145.7 nm, respectively (*p* > 0.05) (Fig. 1A). TEM indicated that a similar spherical bilayer shape of milk sEV from BLV-infected and uninfected cattle was observed (Fig. 1B). WB analysis successfully detected milk sEV-surface-marker CD63 and internal protein HSP70 from BLV-infected and uninfected cattle (Fig. 1C and Supplementary Fig. 3).

**Figure 3:**
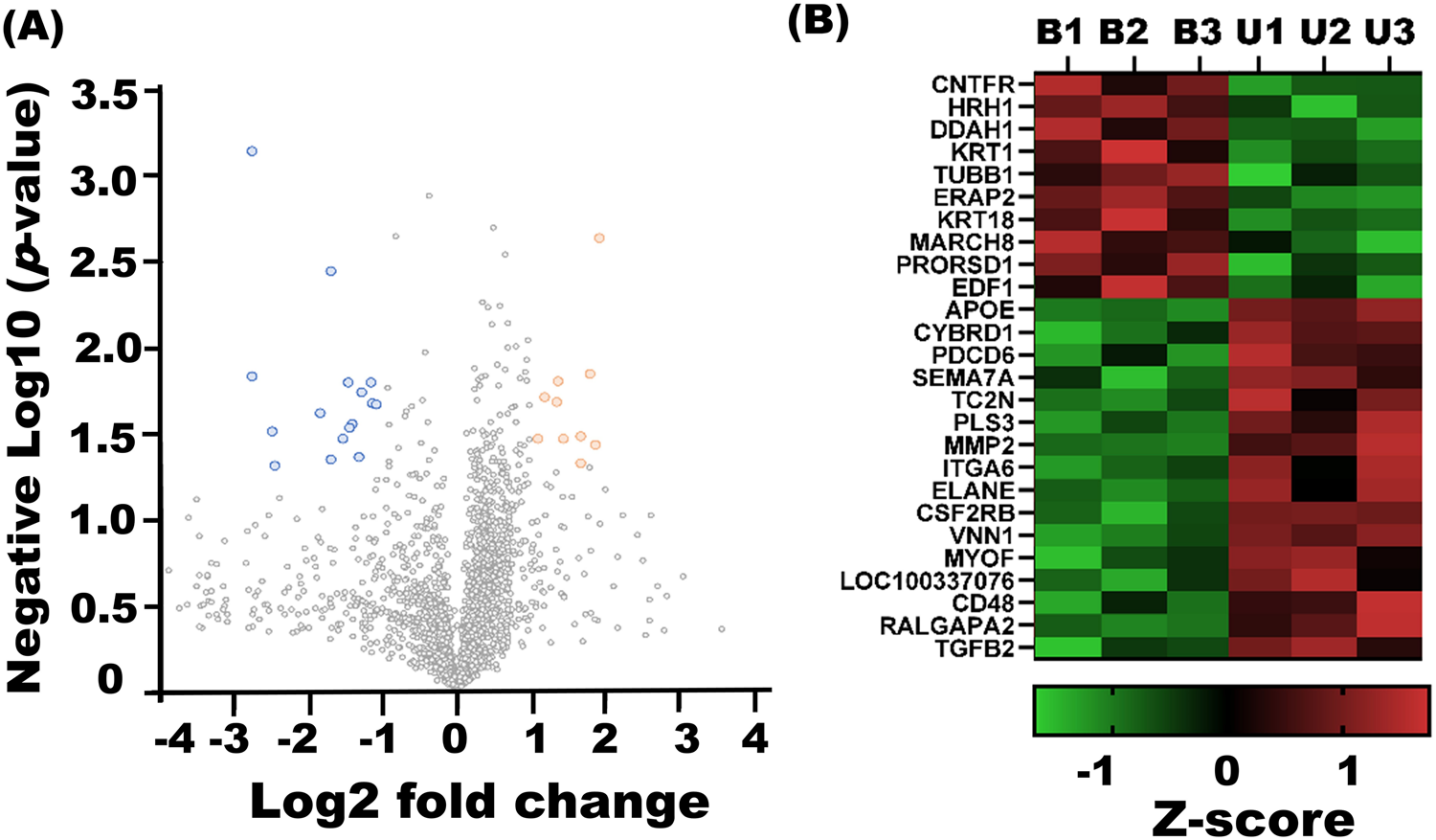
Volcano plot demonstrated the expression of milk sEV proteins from BLV-infected and uninfected cattle. Up/down-regulated sEV proteins were indicated in orange and blue colours, respectively. Proteins not classified as up/down-regulated were plotted in grey colour. X-axis and Y-axis indicated a more than two-fold change (in log2 scale) and − log10 with significance (*p* < 0.05) (**A**). Heat map of hierarchical clustering indicated the candidates for differentially expressed proteins milk sEV from BLV-infected cattle. Up/down-regulated proteins were indicated by red/green colour, respectively (**B**). GraphPad Prism software, version 8 (https://www.graphpad.com) was used to generate the volcano plot and heat map.

### Proteomic profiling of milk sEV

Isolated sEV were analysed using nano LC–MS/MS method to compare the milk sEV proteins of BLV-infected cattle with that of uninfected cattle. The proteomic analysis revealed a large number of proteins in milk sEV from both BLV-infected and uninfected cattle (Table 2). A total of 1330 proteins in milk sEV were found as common proteins among three BLV-infected cattle. Whereas, a total of 1512 proteins in milk sEV were found as common proteins among three uninfected cattle. From the result, a total of 1212 proteins in milk sEV were overlapped after comparing BLV-infected cattle with those of uninfected cattle (Fig. 2). Most significantly, our study identified a total of 118 proteins that were the candidates for uniquely expressed proteins in milk sEV from BLV-infected cattle (Supplement Table 1). The volcano plot indicated the differential encapsulation of milk sEV proteins between BLV-infected and uninfected cattle (Fig. 3A). The heat map showed that a total of 26 proteins in milk sEV showed more than two-fold up/down-regulation with a significant difference (*p* < 0.05) in BLV-infected cattle (Fig. 3B and Table 3).

**Table 2:** Number of proteins identified in milk sEV from three BLV-infected and three uninfected cattle.

Proteins	BLV-infected cattle			Uninfected cattle		
	B1	B2	B3	U1	U2	U3
Identified proteins	1549	1478	1555	1560	1974	1690
Common proteins	1330			1512

**Table 3:** The candidates for differentially expressed proteins in milk sEV from BLV-infected cattle.

UniPort accession	Protein name	Gene name	Regulation
A0A3Q1LQY1	Ciliary neurotrophic factor receptor	CNTFR	UP
P30546	Histamine H1 receptor	HRH1	UP
P56965	N(G),N(G)-dimethylarginine dimethylaminohydrolase 1	DDAH1	UP
G3N0V2	Keratin 1	KRT1	UP
A0A3Q1M442	Tubulin beta chain	TUBB1	UP
A6QPT7	Endoplasmic reticulum aminopeptidase 2	ERAP2	UP
F6S1Q0	Keratin 18	KRT18	UP
Q0VD59	E3 ubiquitin-protein ligase MARCH8	MARCH8	UP
A1A4Q2	Prolyl-tRNA synthetase associated domain-containing protein 1	PRORSD1	UP
Q3T0V7	Endothelial differentiation-related factor 1	EDF1	UP
Q03247	Apolipoprotein E	APOE	Down
F1MLZ1	Cytochrome b reductase 1	CYBRD1	Down
A0A3Q1LF77	Programmed cell death 6	PDCD6	Down
A0A3Q1NI92	Semaphorin 7A	SEMA7A	Down
E1BEH7	Tandem C2 domains, nuclear	TC2N	Down
A7E3Q8	Plastin-3	PLS3	Down
Q9GLE5	72 kDa type IV collagenase	MMP2	Down
A0A3Q1M8K4	Integrin subunit alpha 6	ITGA6	Down
A6QPP7	ELA2 protein	ELANE	Down
F1MXH7	Colony stimulating factor 2 receptor beta common subunit	CSF2RB	Down
Q58CQ9	Pantetheinase	VNN1	Down
F1N3I4	Myoferlin	MYOF	Down
G3MW08	Uncharacterized protein	LOC100337076	Down
Q2KHZ6	CD48 molecule	CD48	Down
A0A3Q1LJ82	Ral GTPase activating protein catalytic alpha subunit 2	RALGAPA2	Down
P21214	Transforming growth factor beta-2 proprotein	TGFB2	Down

### Functional and protein–protein interaction (PPI) network analysis

**Figure 4:**
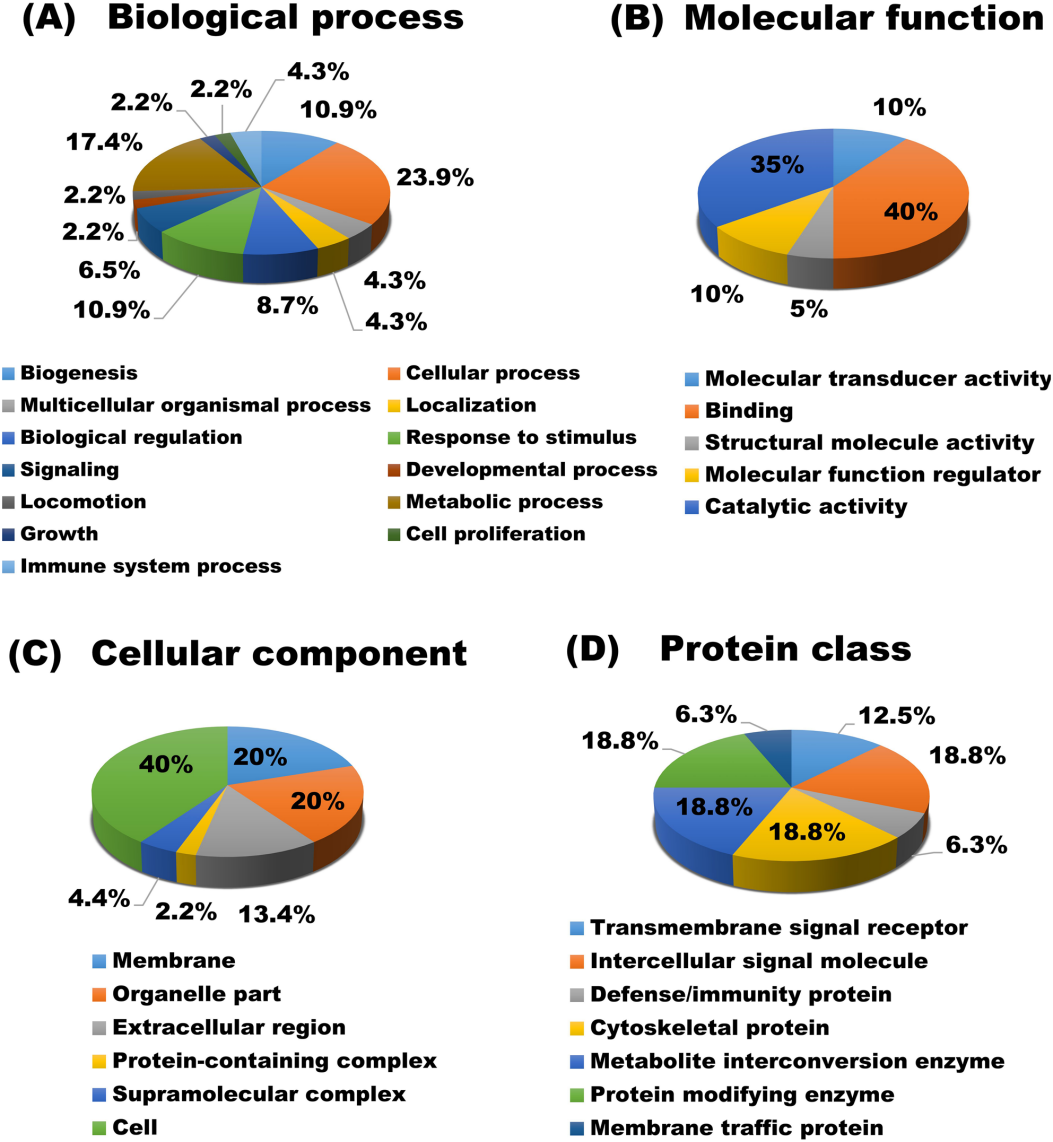
GO analysis of the candidates for differentially expressed proteins in milk sEV from BLV-infected cattle. The candidates for differentially expressed proteins in milk sEV from BLV-infected cattle were analysed using Panther software and categorised according to the biological process (**A**), molecular function (**B**), cellular component (**C**), and protein class (**D**).

The GO analysis was performed to evaluate the candidates for uniquely (Supplementary Fig. 4A-4D) or differentially expressed proteins (Fig. 4A–4D) to obtain a comprehensive image of the changes in proteins in milk sEV from BLV-infected cattle. The candidates for uniquely or differentially expressed proteins in milk sEV were engaged in a broad range of biological processes such as cellular process, metabolic processes, response to stimulus, signalling, biogenesis, and so on. As for the molecular function, the majority of the proteins appeared to participate in binding and catalytic activity. In the cellular component category, proteins were mainly localised in the cell, membrane, and organelles. Furthermore, the majority of the proteins were classified as cytoskeletal proteins, protein modifying enzymes, translational proteins, and metabolite interconversion enzyme.

**Figure 5:**
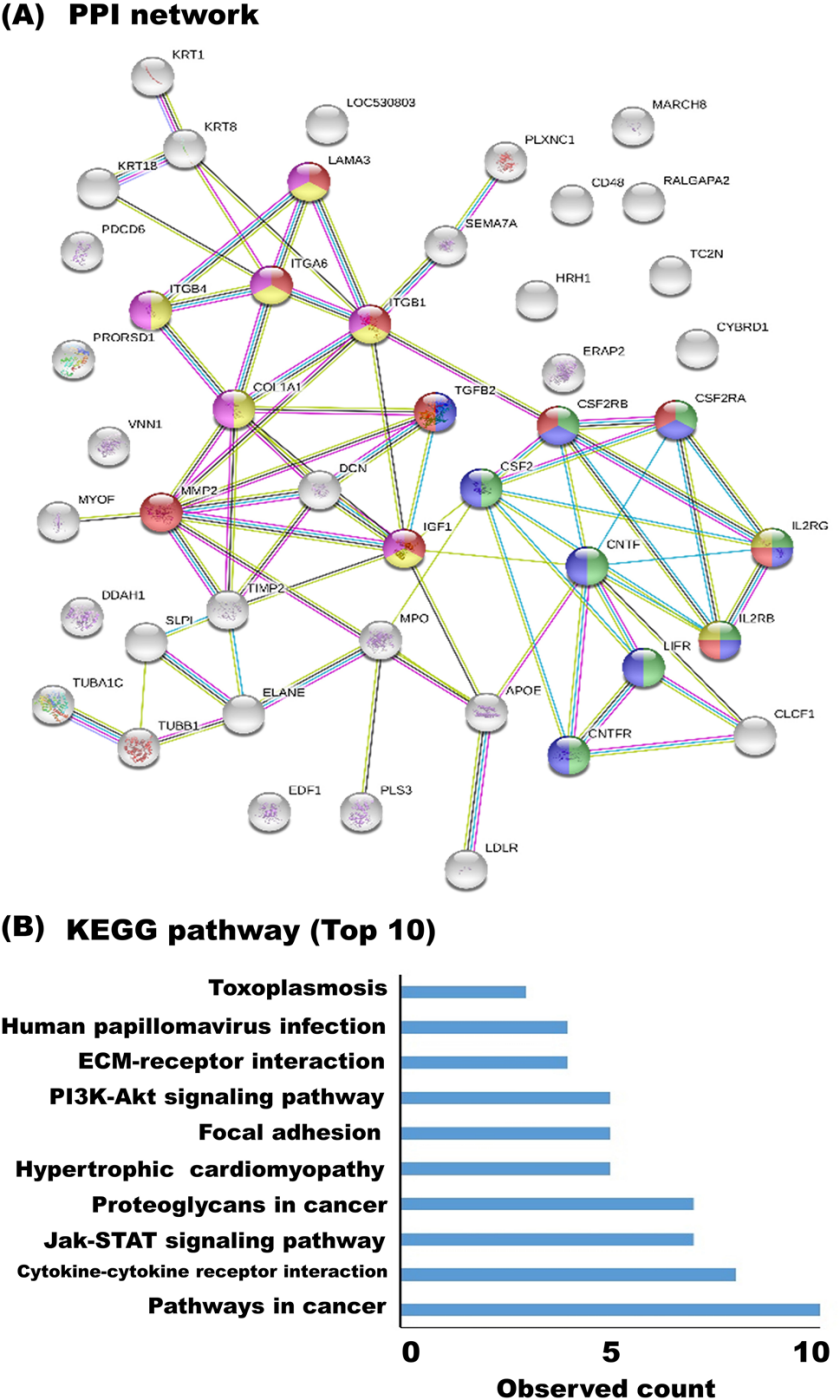
PPI network and KEGG pathway analysis. The PPI network was analysed by STRING software to evaluate the candidates for differentially expressed proteins in milk sEV from BLV-infected cattle (**A**). In the network, nodes and edges indicated milk sEV proteins and physical and/or functional interactions of the milk sEV proteins, respectively. Empty nodes represent the proteins of unknown three-dimensional structure, and filled nodes represent the proteins with some known or predicted three-dimensional structure. Different coloured lines between the proteins represent the various types of interactions in STRING (databases, experiments, neighbourhood, gene fusion, co-occurrence, text mining, co-expression, and homology). The candidates for differentially expressed proteins in milk sEV from BLV-infected cattle were analysed using KEGG software, and the top 10 KEGG pathways were demonstrated in the bar diagram (**B**).

The candidates for uniquely or differentially expressed proteins in milk sEV were illustrated in the protein–protein interaction (PPI) network by STRING analysis. Importantly, the unique proteins in milk sEV have been illustrated a connectivity PPI network (Supplementary Fig. 5A) relating to several KEGG pathways including ribosome, pathways in cancer, regulation of actin cytoskeleton, chemokine signalling pathway, focal adhesion, and so on (top 10 KEGG pathways were shown in Supplementary Fig. 5B). The candidates for differentially expressed proteins in milk sEV made a strong cluster of networking in PPI (Fig. 5A) relating to several KEGG pathways including pathways in cancer, cytokine-cytokine receptor interaction, JAK-STAT signalling pathways, proteoglycans in cancer, focal adhesion, and so on (top 10 KEGG pathways were shown in Fig. 5B).

## Discussion

In this study, milk sEV were characterized by NTA, TEM, and WB analyses. The results by NTA, TEM and WB analyses were indicating the typical definition and enrichment of sEV according to the MISEV2018 guidelines^3^. Proteomic analysis revealed a total of 1330 proteins in milk sEV were common among three BLV-infected cattle, of which 118 proteins were the candidates for uniquely expressed proteins compared to those from uninfected cattle. The identified protein numbers in milk sEV from BLV-infected cattle were distinctive and relatively larger to those reported in previous studies^9,17–19^. These results indicated that our current study was efficiently isolated milk sEV from BLV-infected cattle which allowed the protein count in milk sEV to increase. Moreover, nanoLC-MS/MS method had detected of low-abundance compounds in small amount of proteins resulting a high-throughput protein count in milk sEV from BLV-infected cattle^20^.

Although the mortality rate is low, the most noticeable negative outcome of BLV infection is that cattle develop to lymphosarcoma. However, it is difficult to suspect and detect which BLV-infected cattle could be developed with lymphosarcoma soon or later. Collection and analysis of milk sEV proteins hold to promise as a novel monitoring platform for BLV infection of cattle. Though sEV play a vital role in cell to cell communication^7^ and influence the physiological processes^8^, their function in infectious diseases is still under progress. Huang et al.^21^ reported that *Staphylococcus aureus* modified the protein expression in mammary tissue of cattle indicating that infectious diseases are capable of changing the normal proteomic profile of infected tissues. Recently, we demonstrated that mRNAs profile in milk sEV from BLV-infected cattle were altered compared with those of uninfected cattle^15^. However, to date, no research has been performed on identifying the changes of proteins in milk sEV from BLV-infected cattle. Therefore, the current study was conducted to evaluate the proteins encapsulation in milk sEV from BLV-infected cattle.

The present study detected a total of 26 proteins which were the candidates for differentially expressed proteins in milk sEV from BLV-infected cattle compared with those of uninfected cattle. Previous studies have been identified different types of proteins from blood or tumour tissues that were feasible in understanding the tumour progression in human. Some of the up/down-regulated proteins in our present study such as CNTFR, DDAH1, TUBB1, MARCH8, KRT1, KRT18, APOE, PDCD6, MMP2, ITGA6, MYOF, and TGFB2 were previously reported to be associated with certain tumorigenesis and progression in malignancies^22–33^. Taken together, these results indicated that as an oncogenic viral disease in cattle, BLV infection could alter the encapsulated sEV proteins; therefore, the new proteins are appearing within milk sEV. The results also suggested that these proteins could be crucial in obtaining information regarding BLV infection and its pathogenesis in cattle.

GO and KEGG analyses identified several functional terms that were enriched by the candidates for uniquely or differentially expressed proteins in milk sEV. Attention was paid to the aforementioned milk sEV proteins to unveil diverse GO terms and numerous KEGG pathways for BLV infection. In the biological process, where proteins were predominantly associated with the cellular process, metabolic process, response to stimulus, and developmental process suggesting cellular proliferation, alteration, and attachment, extensively participated during BLV infection. Binding and catalytic activity were the most prevalent molecular functions, which indicated that direct regulation of protein–protein interactions might be the key process during BLV infection and its pathogenicity in the host. The cellular component indicated that the identified proteins were enriched in the cell and membrane, thus suggesting that the contents of sEV shared close ties with their host cells. The KEGG analysis revealed that the candidates for uniquely or differentially expressed proteins involved in many pathways including pathways in cancers, cytokine-cytokine interaction, PI3K-Akt signalling pathway, Jak-STAT signalling pathway, and focal adhesion. Several of these KEGG pathways played an important role in tumour cell proliferation, metastasis, leukemogenesis as well as formation of solid tumours reported previously in human^34^. The results suggested that the biologically important pathways are probably involved in development and/or progression of BLV-induced leukemogenesis and tumour formation in cattle. Previous studies reported that mRNAs in milk and blood from BLV-infected cattle involved in diverse biological functions and many other pathways that are consistent with our current study^15,35^.

In conclusion, proteomic analysis identified a total of 1330 common proteins in milk sEV from BLV-infected cattle, from which 118 proteins were the candidates for uniquely expressed proteins compared with those of uninfected cattle. Besides, GO and KEGG analyses showed a new biological phenomenon and distinct pathways that may have a great contribution into the BLV pathogenesis. The results of this study could be solid ground to facilitate future development of milk sEV-based disease monitoring. This is the first study to present the proteomic analysis of milk sEV from BLV-infected cattle that could have wide applicability in molecular biology.

In this study, since verification of the data by ELISA or other, equivalent means has not been performed, further studies are required.

## Materials and methods

All experiments were performed in accordance with relevant guidelines and regulations of the Gifu University Animal Care and Use Committee (approval number 17046 and approved on 4 September, 2017). Additionally, experiments using cows were carried out in compliance with the standards of animal rights, welfare, and with minimum distress by following relevant guidelines and regulations of the Gifu University Animal Care and Use Committee.

### Blood samples

Blood samples of 10 ml of each of the 16 Holstein cows were collected from two different farms and directly allocated to vacuum blood collection tubes with or without an anti-coagulant (VP-AS076K, VP-NA050K, and VP-H070K, Terumo, Tokyo, Japan). Total WBCs and lymphocyte counts were measured by an automatic cell counter Celltac α (Nihon Kohden, Tokyo, Japan). The increased lymphocyte count was checked based on the European Community’s leukosis key^16^. After WBCs and lymphocyte counts, 1.3 ml of each of the anticoagulated blood samples were centrifuged at 2500 × *g* for 15 min at 25 °C for plasma separation by a centrifuge, MAX-307 (Tomy Seiko, Tokyo, Japan). Plasma samples were collected from the top portion of the tube and used for lactate dehydrogenase (LDH) isozymes measurement later.

### Detection of antibodies

Serum was separated from blood by centrifugation at 3000 × *g* for 15 min at 25 °C by using a centrifuge, MAX-307. Anti-BLV antibodies in serum were measured by using an anti-BLV antibody ELISA kit (JNC, Tokyo, Japan) according to the manufacturer’s instructions.

### Detection of BLV

WBC was isolated from blood by hemolysis of red blood cells with 0.83% ammonium chloride followed by washing twice with phosphate buffer saline (PBS). Total amount of DNA was extracted from WBCs by using QIAamp DNA Mini Kit (51304, Qiagen, Hilden, Germany) according to the manufacturer’s instructions. After measurement of DNA concentration of WBC DNA by a spectrophotometer NanoDropLite (Thermo Fisher Scientific, Waltham, MA, USA). Primers to amplify the envelope or pX region of BLV were used for nested PCR according to the protocols by Fechner et al.^36^ and Murakami et al.^37^. PCR was carried out in a total reaction volume of 20 µl containing 0.5 U of polymerase from GoTaq Hot Start Green Master Mix (M5122, Promega, Madison, WI, USA) or SapphireAmp Fast PCR Master Mix (RR350A, Takara Bio, Kusatsu, Japan), 0.5 µM of forward and reverse primers, and 1 µl of extracted WBC DNA (100–400 ng). Thermal cycling condition was as follows: 95 °C for 2 min, followed by 35 cycles of 94 °C for 45 s, 62 °C for 30 s, 72 °C for 30 s, and finally 72 °C for 4 min.

### Measurement of proviral load

BLV-infected cattle with HPL in blood were selected for this study. It was reported that BLV-infected cattle with HPL in blood were considered as cattle at high risk to be BLV spreaders and might be one of the factors of disease progression^38^. BLV proviral load was measured by using 100 ng of WBC DNA by a quantitative real-time PCR (qRT-PCR). The amplification was carried out in a reaction mixture containing 10 µl of THUNDERBIRD Probe qPCR Mix (A4250K, Toyobo, Osaka, Japan), 0.3 µl of CoCoMo-BLV Primer/Probe (A803, Riken Genesis, Tokyo, Japan), 5 µl of a template DNA sample, and PCR grade water to increase the volume up to 20 µl. For the proviral quantification, BLV BoLA-DRA gene Plasmid DNA was used from the kit (A804, Riken Genesis) and BLV proviral DNA was measured by a Thermal Cycler Dice Real Time System III (TP970, Takara Bio) according to the manufacturer’s instructions. After the measurement, BLV proviral copies of > 30,000 in 10^5^ WBCs DNA was considered as HPL in BLV-infected cattle (Table 1). Hematological test, detection of serum antibodies against BLV, detection of BLV provirus, and measurement of BLV proviral load were conducted by the Gifu Central Livestock Hygiene Service Center (Gifu, Japan).

### Measurement of lactate dehydrogenase (LDH) isozymes

A previous study reported that LDH activities in the serum, mainly, increased LDH 2 and 3 isozyme percentages, reflected progression of EBL, thereby making it a key parameter for the diagnosis of lymphosarcoma^39^. Therefore, we also focused on serum LDH isozyme activity. BLV-infected cattle with LDH 2 + 3 > 30% was selected. LDH isozymes were measured by a Hydrasys 2 Scan (Sebia, Paris, France) using HYDRAGEL 7 ISO-LDH (Sebia), which was conducted by a clinical laboratory testing company, Fujifilm Vet Systems (Tokyo, Japan).

### Collection of milk samples

Milk samples were collected from three BLV-infected and three uninfected healthy cattle. After collection, both milk samples were transported quickly to the laboratory in a cool box to maintain the temperature and were stored at 4 °C for further use.

### Isolation of sEV

For the isolation of milk sEV from BLV-infected and uninfected cattle, we followed the procedure previously described by Yamauchi et al.^40^ and Rahman et al.^41^ with slight modifications. Importantly, after defatting of milk, milk sEV were purified using acetic acid followed by sequential filtration through 1.0, 0.45, and 0.2 μm filters (GA-100, C045A047A, and C020A047A, Advantec, Tokyo, Japan). Subsequently, milk sEV were concentrated by ultracentrifugation (UC) at 100,000 × *g* at 4 °C for 1 h using a P42A angle rotor (Hitachi Koki, Tokyo, Japan) in a Himac CP80WX ultracentrifuge (Hitachi Koki). After the first UC, the supernatant was discarded and the pellet was resuspended with PBS up to 10 ml into a 13PET tube (Hitachi Koki) followed by another UC at 100,000 × *g* at 4 °C for 1 h using a P40ST swing rotor (Hitachi Koki). sEV pellet was collected and resuspended with 100 µl of PBS for further use.

### Protein concentration of milk sEV

Protein concentration of recovered sEV were estimated by Lowry’s method^42^ using a DC protein assay kit (500-0113, 500-0114, 500-0115, and 5,000,007; Bio-Rad Laboratories, Hercules, CA, USA) with a spectrophotometer, GeneQuant100 (GE Healthcare, Chicago, IL, USA).

### Isolation of RNA and detection of BLV genomic RNA and BLV proteins in milk sEV

Total RNA was extracted from milk sEV from BLV-infected cattle by using QIAamp viral RNA Mini Kit (52906, Qiagen, Hilden, Germany) according to the manufacturer’s instructions. The RNA concentration was measured by a spectrophotometer NanoDropLite (Thermo Fisher Scientific). cDNA was synthesis by reverse transcription reaction using 5 × PrimeScript RT Master Mix (RR036A-1, Takara Bio) followed by nested PCR was performed to amplify the envelope or pX region of BLV^36,37^. WB analysis was performed as described in a previous study^41^. After gel electrophoresis and trans-blotting, membranes were blocked with 5% non-fat skim milk in Tris-buffered saline [0.1 M Tris–HCl (pH 8.0) and 0.03 M NaCl] containing 0.1% Tween-20 (TBST) at room temperature (RT) for 30 min. For the detection of BLV proteins gp51 in milk sEV from BLV-infected cattle, monoclonal antibody specific to a D-D' epitope on BLV gp51 (Env) (1:400, VMRD, Pullman, WA, USA) was used. The membrane was incubated for 1 h at RT, diluted in 1% non-fat skim milk in TBST, followed by washing thrice with TBST. The secondary antibody, anti-mouse IgG sheep antibody (1:1000, NA9310, GE Healthcare, Little Chalfont, UK) conjugated with horseradish peroxidase, were diluted with TBST incubated for 1 h at RT followed by washing thrice with TBST. Peroxidase activity was detected using a Pierce ECL Plus substrate (Thermo Fisher Scientific) and visualised by using a chemiluminesence apparatus (ChemiDoc XRS + , Bio-Rad Laboratories).

### Characterization of milk sEV

NTA analysis of milk sEV from BLV-infected and uninfected cattle was performed using a NanoSight LM10V-HS, NTA 3.4 instrument by an assigning company (Quantum Design Japan, Tokyo, Japan). Morphological examination of isolated milk sEV from BLV-infected and uninfected cattle was carried out by TEM as described in a previous study^41^ with slight modifications. The sEV pellet was diluted 10 times from its original concentration with distilled water, applied to glow-discharged carbon support films on copper grids followed by stained with 2% uranyl acetate. The samples were then visualized under an electron microscope, JEM-2100F (JEOL, Tokyo, Japan) at 200 kV. WB analysis was carried out as described in a previous study^41^ with slight modifications. The primary antibodies, anti-CD63 (1:400, M-13, SC-31214, Santa Cruz Biotechnology, Santa Cruz, CA, USA) or anti-HSP70 (1:100, N27F3-4, Enzo Life Science, Farmingdale, NY, USA) following the secondary antibodies, anti-goat IgG donkey antibody (1:2000, SC-3851, Santa Cruz Biotechnology) or anti-mouse IgG sheep antibody (GE Healthcare) conjugated with horseradish peroxidase, were used as described above.

### Proteomic and Scaffold Data Independent Acquisition (Scaffold DIA) analysis

For the proteomic profiling, all milk sEV samples were analyzed by the nanoLC-MS/MS method (UltiMate 3000 RSLCnano System, Thermo Fisher Scientific) as described previously by Nguyen et al.^43^ with slight modifications. All procedures of proteomic analysis were performed by an entrusted company, Hakarel (Osaka, Japan). Accession number of each sEV protein was obtained from the UniPort database for Bos taurus (https://www.uniprot.org/ proteomes/UP000009136). The results were further analyzed by the Scaffold DIA software (Proteome Software, Portland, OR, USA) to compare the peptide counts of the identified proteins, considering the false discovery rate > 1%. A moderated t-test with Benjamini–Hochberg test^44^ were performed to assess the statistical significance of the data. Corrected *p*-value cut off of 0.05 was applied. From our data sets, common proteins present in all three samples from BLV-infected and those from three uninfected cattle were considered for listing up and the proteins without a gene name or those with a dual gene name were excluded.

### Functional and STRING interaction network analysis

GO and KEGG pathway^45^ analyses were performed using the Database for Annotation, Visualization and Integrated Discovery (DAVID version 6.8) (https://david.ncifcrf.gov/)^46^. The biological process, molecular function, cellular component, and protein class were determined using Panther software, online version (http://www.pantherdb.org/). An adjusted *p*-value of less than 0.05 was considered statistically significant. In this study, we used the Search Tool for the Retrieval of Interacting Genes (STRING) database, version 10.5 (https://string-db.org) to construct the interaction network of the candidates for uniquely or differentially expressed proteins in milk EVs from BLV-infected cattle.

### Statistical analysis

Statistical analysis was performed using the Student's t-test to compare the milk sEV proteins from BLV-infected and uninfected cattle. The *p* value 0.05 was considered as statistically significant.

## Supplementary Information

Supplementary Legends.

Supplementary Information

## Data Availability

Raw data of proteomic analysis of milk sEV from BLV-infected and uninfected cattle was deposited in the Mendeley data repository (Direct URL: Mendeley Data, V1, https://doi.org/10.17632/zxb5vhjrf 5.1 and https://doi.org/10.17632/7c2ddgwcgt.1).
