# Supplementary material for: Proteomic profiling of milk small extracellular vesicles from bovine leukemia virus-infected cattle
Source: Sci Rep. 2021 Feb 3;11:2951. doi: 10.1038/s41598-021-82598-2 (PMC7858626; doi:10.1038/s41598-021-82598-2)
Supplement: Supplementary file 1 — Supplementary Legends. [file 41598_2021_82598_MOESM1_ESM.docx]

**Additional Information**

Supplementary Figure 1. Protein concentration of milk sEV from BLV-infected and uninfected cattle. There is not significant difference present among these group (*p*>0.05). Supplementary Figure 2. Reverse transcription-nested PCR and WB analysis. BLV genomic RNA was not detected in milk sEV from BLV-infected cattle by reverse transcription-nested PCR analysis (A). BLV protein gp51 was not detected in milk sEV from BLV-infected cattle by WB analysis (B). Supplementary Figure 3. Milk sEV surface protein and internal protein was successfully detected by using anti-CD63 and -HSP70 antibodies by WB analysis. Supplementary Figure 4. GO analysis of the candidates for uniquely expressed proteins in milk sEV from BLV-infected cattle. GO of the candidates for uniquely expressed proteins in milk sEV from BLV-infected cattle were analysed by Panther software and results were categorised according to the biological process (C), molecular function (D), cellular component (E), and protein class (F). Supplementary Figure 5. STRING protein-protein interaction network (A) and KEGG pathway (top 10 KEGG pathways) analysis of the candidates for uniquely expressed proteins in milk sEV from BLV-infected cattle were demonstrated in the bar diagram (B) Supplementary Table 1. The candidates for uniquely expressed proteins in milk sEV from BLV-infected cattle.
