# Supplementary material for: Proteomic profiling of milk small extracellular vesicles from bovine leukemia virus-infected cattle
Source: Sci Rep. 2021 Feb 3;11:2951. doi: 10.1038/s41598-021-82598-2 (PMC7858626; doi:10.1038/s41598-021-82598-2)
Supplement: Supplementary file 2 — Supplementary Information [file 41598_2021_82598_MOESM2_ESM.docx]

**Proteomic profiling of milk small extracellular vesicles from bovine leukemia virus-infected cattle**

Md. Matiur Rahman^1,2,3^, Shigeo Takashima^4^, Yuji O. Kamatari^5^, Yassien Badr^2,6^, Yuko Kitamura^7^, Kaori Shimizu^2^, Ayaka Okada^2,8^, Yasuo Inoshima^1,2,8,9*^

^1^The United Graduate School of Veterinary Sciences, Gifu University, 1-1 Yanagido, Gifu, Gifu 501-1193, Japan; ^2^Laboratory of Food and Environmental Hygiene, Cooperative Department of Veterinary Medicine, Gifu University, 1-1 Yanagido, Gifu, Gifu 501-1193, Japan; ^3^Department of Medicine, Faculty of Veterinary, Animal and Biomedical Sciences, Sylhet Agricultural University, Sylhet-3100, Bangladesh; ^4^Division of Genomics Research, Life Science Research Center, Gifu University, 1-1 Yanagido, Gifu, Gifu 501-1193, Japan; ^5^Division of Instrumental Analysis, Life Science Research Center, Gifu University, 1-1 Yanagido, Gifu, Gifu 501-1193, Japan; ^6^Department of Animal Medicine (Branch of Infectious Diseases), Faculty of Veterinary Medicine, Damanhour University, El-Beheira, Egypt; ^7^Gifu Prefectural Chuo Livestock Hygiene Service Center, 1-1 Yanagido, Gifu, Gifu 501-1112, Japan; ^8^Education and Research Center for Food Animal Health, Gifu University (GeFAH), 1-1 Yanagido, Gifu, Gifu 501-1193, Japan; ^9^Joint Graduate School of Veterinary Sciences, Gifu University, 1-1 Yanagido, Gifu, Gifu 501-1193, Japan.

*Correspondence to: Y. Inoshima; Gifu University, 1-1 Yanagido, Gifu, Gifu 501-1193, Japan.

Email: inoshima@gifu-u.ac.jp


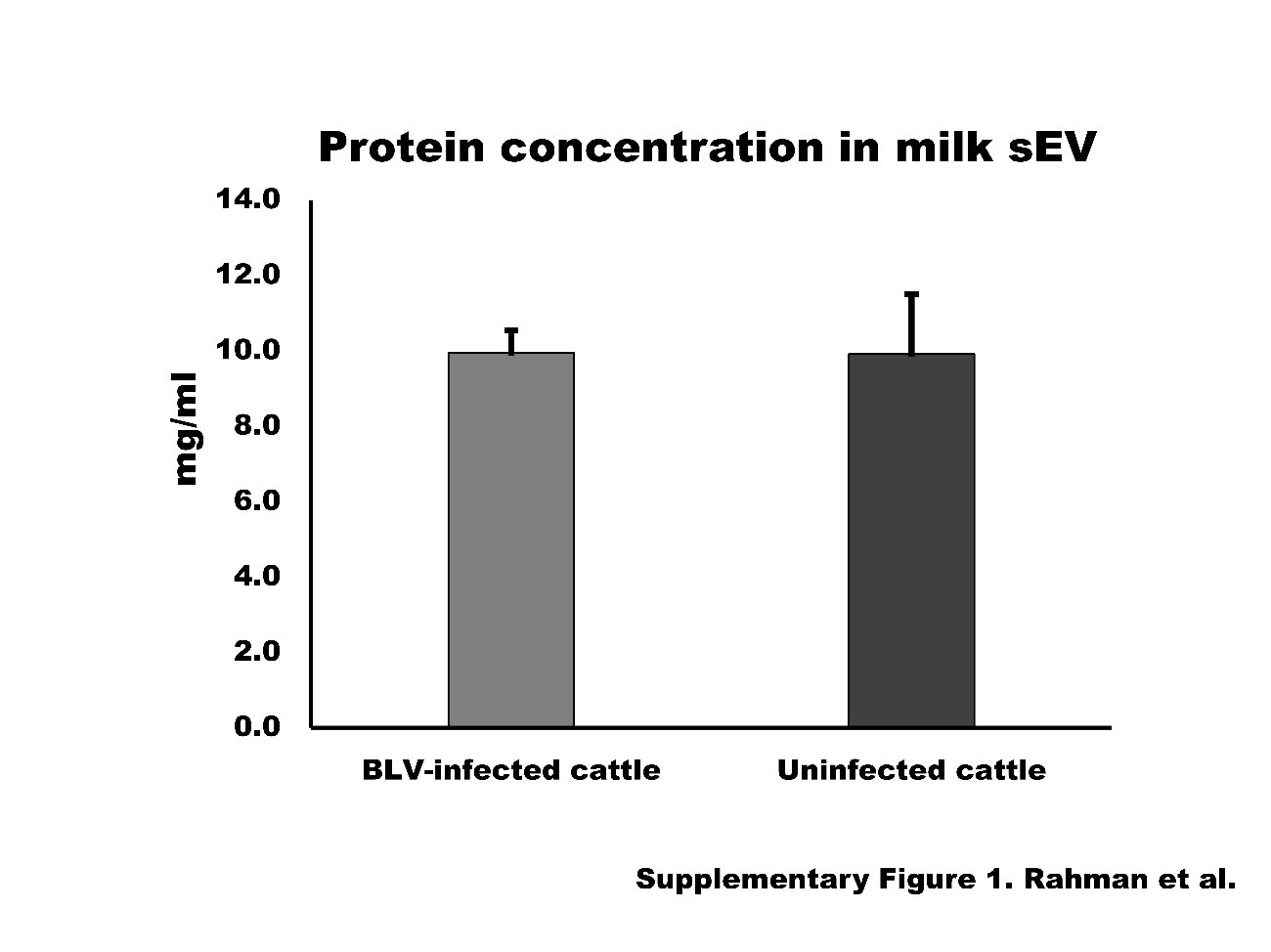


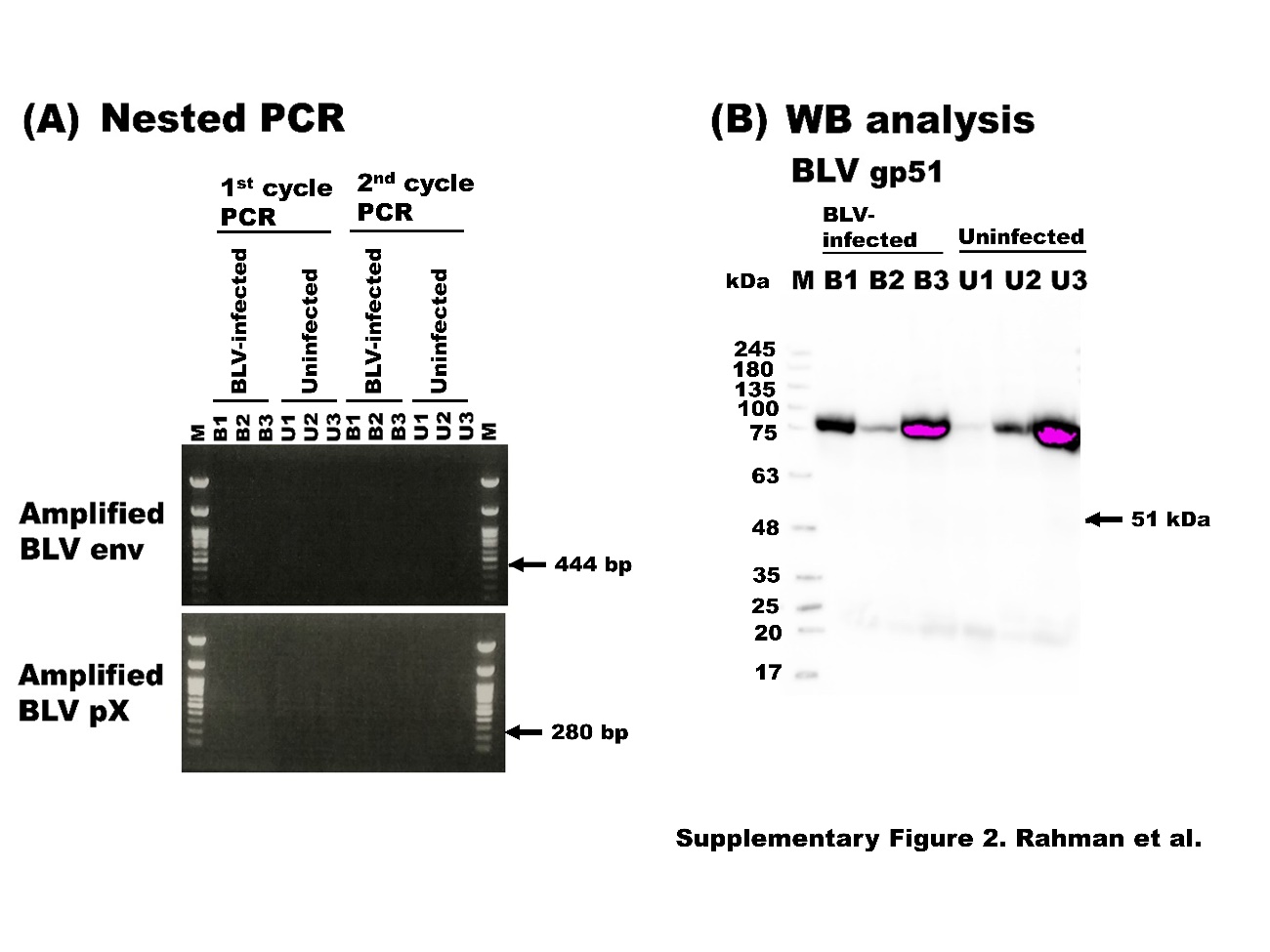


**
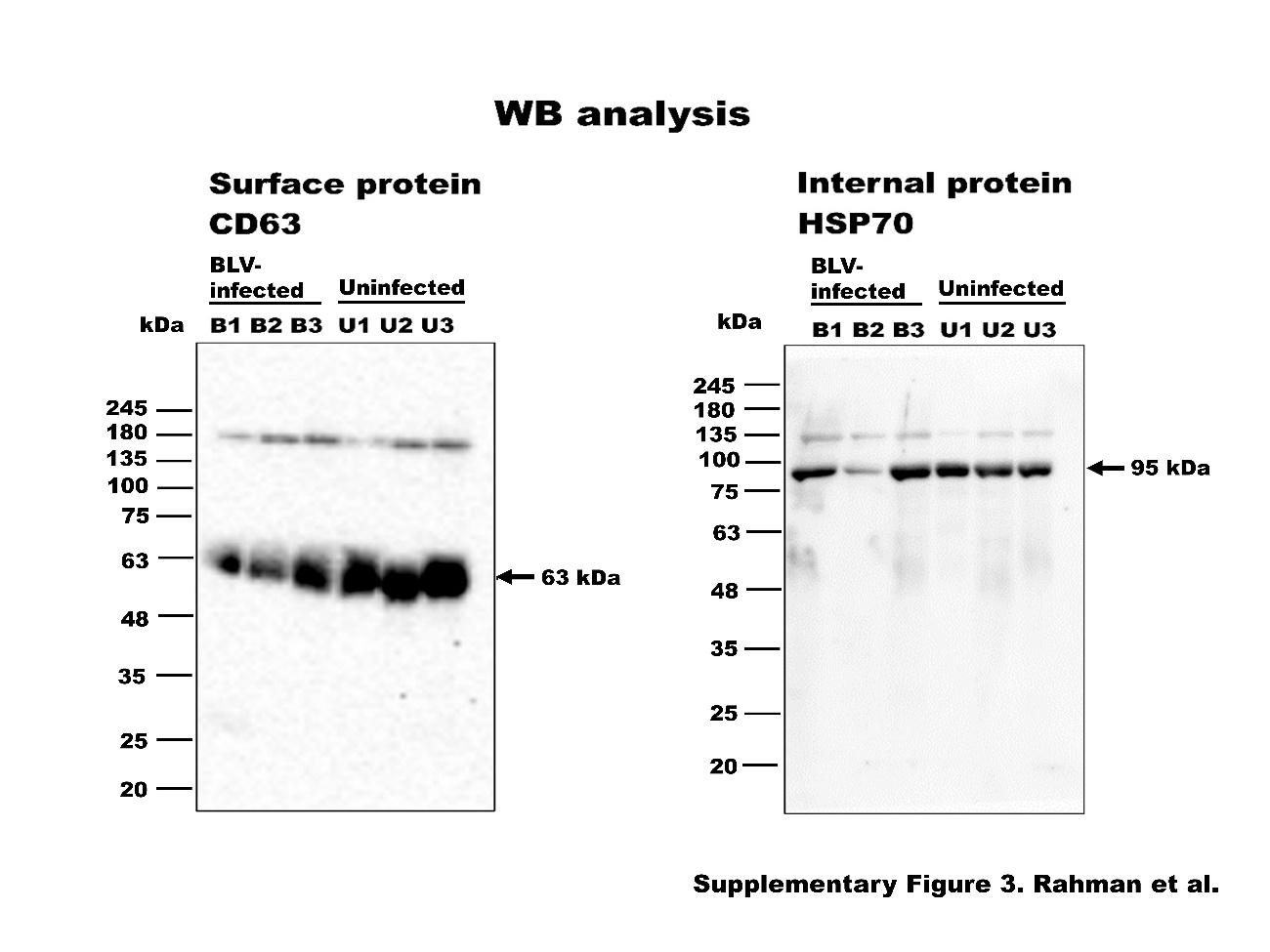
**


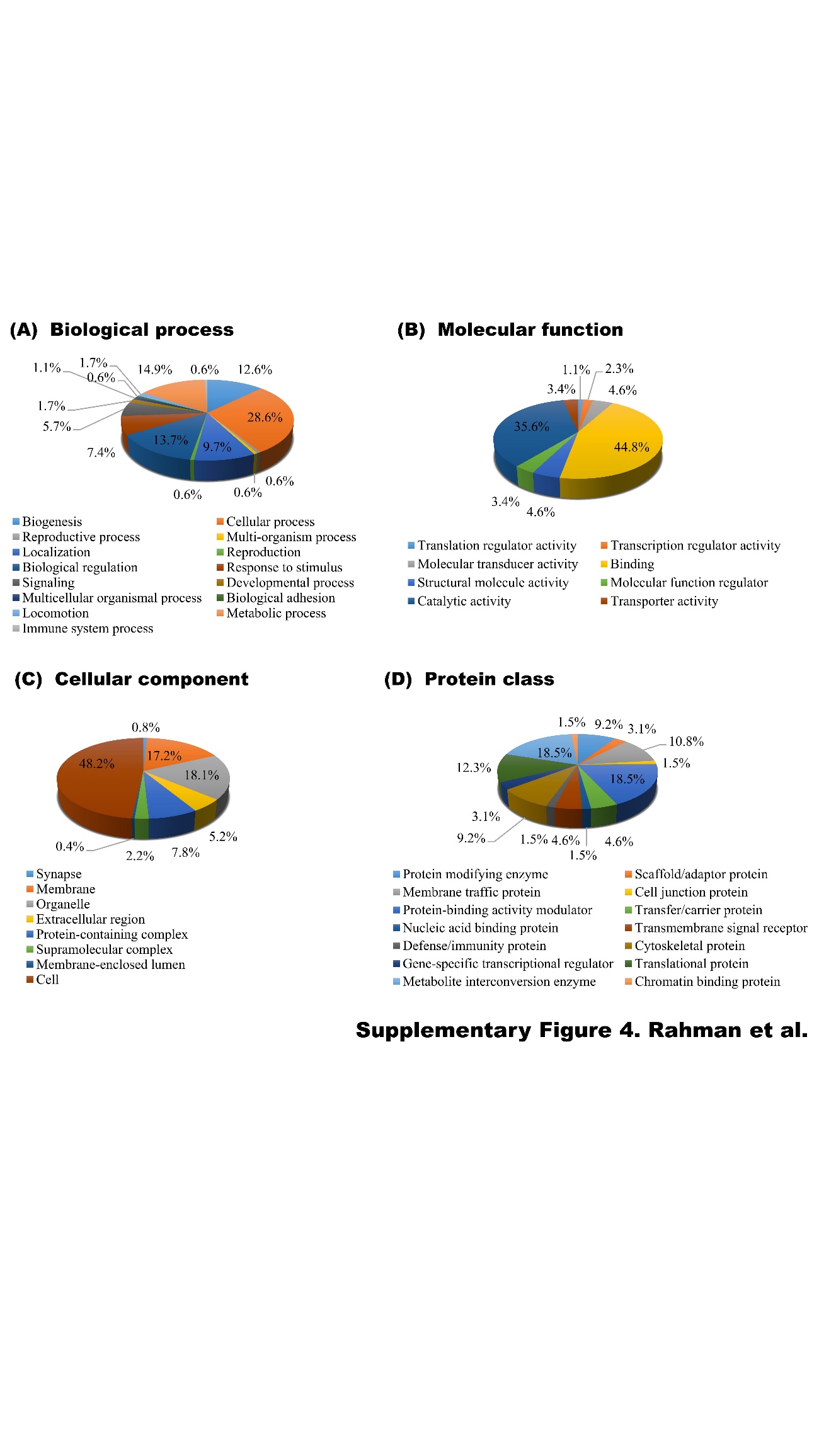


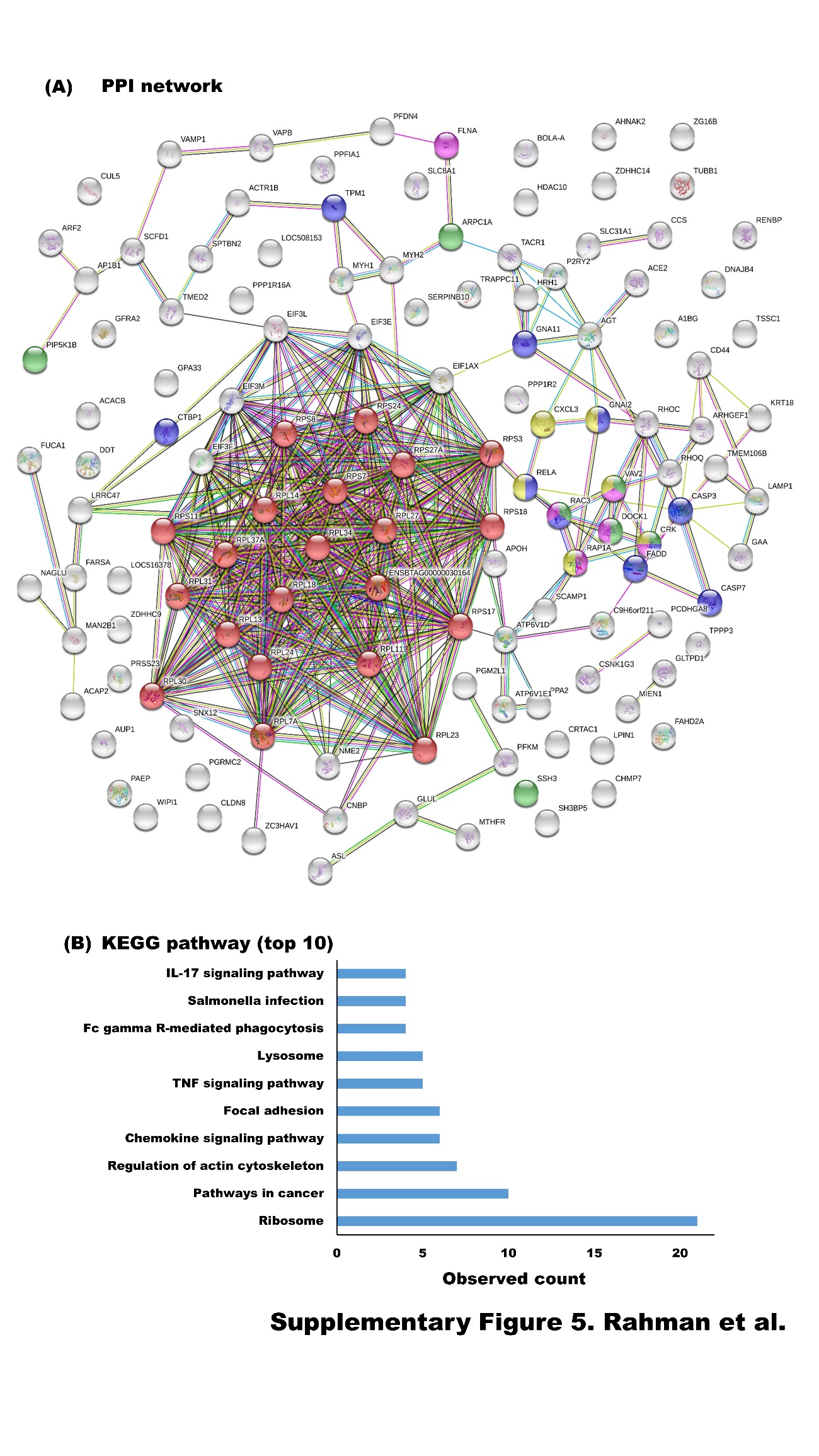


**Supplementary Table 1. The candidates for uniquely expressed proteins in milk sEV from BLV-infected cattle**

| **UniPort accession no.** | **Protein name** | **Gene name** |
| --- | --- | --- |
| Q2KJF1 | Alpha-1B-glycoprotein | A1BG |
| F1MSC3 | Acetyl-CoA carboxylase beta | ACACB |
| A0A3Q1M1X6 | ArfGAP with coiled-coil, ankyrin repeat and PH domains 2 | ACAP2 |
| Q58DD0 | Angiotensin-converting enzyme 2 | ACE2 |
| A4IFE3 | Beta-centractin | ACTR1B |
| A0A3Q1LGY9 | Angiotensinogen | AGT |
| A0A3Q1LV73 | Uncharacterized protein | AHNAK2 |
| A0A3Q1LMB5 | AP complex subunit beta | AP1B1 |
| P17690 | Beta-2-glycoprotein 1 | APOH |
| Q3SZF2 | ADP-ribosylation factor 4 | ARF4 |
| A0A3Q1LFQ5 | Rho guanine nucleotide exchange factor 1 | ARHGEF1 |
| A3KMX8 | Protein-glutamate O-methyltransferase | ARMT1 |
| Q1JP79 | Actin-related protein 2/3 complex subunit 1A | ARPC1A |
| Q3SZJ0 | Argininosuccinate lyase | ASL |
| P39942 | V-type proton ATPase subunit D | ATP6V1D |
| P11019 | V-type proton ATPase subunit E 1 | ATP6V1E1 |
| Q3ZC65 | AUP1, lipid droplet regulating VLDL assembly factor | AUP1 |
| A0A3Q1LRW6 | Uncharacterized protein | BOLA-A |
| F1MY85 | Complement C5a anaphylatoxin | C5 |
| Q08DY9 | Caspase-3 | CASP3 |
| F1MD58 | Caspase 7 | CASP7 |
| E1BE86 | Copper chaperone for superoxide dismutase | CCS |
| Q29423 | CD44 antigen | CD44 |
| A6H704 | CHMP7 protein | CHMP7 |
| A4IFM4 | Claudin | CLDN8 |
| Q3T0Q6 | Cellular nucleic acid-binding protein | CNBP |
| Q0VCQ0 | Ceramide-1-phosphate transfer protein | CPTP |
| E1BQ32 | CRK proto-oncogene, adaptor protein | CRK |
| A0A3Q1LN98 | Cartilage acidic protein 1 | CRTAC1 |
| A0A3Q1LXL6 | Casein kinase I isoform gamma-3 | CSNK1G3 |

**Supplementary Table 1. (continued)**

| **UniPort accession no.** | **Protein name** | **Gene name** |
| --- | --- | --- |
| A0A3Q1M504 | C-terminal binding protein 1 | CTBP1 |
| A0A3Q1MXH0 | Cullin 5 | CUL5 |
| F1MD23 | C-X-C motif chemokine | CXCL3 |
| Q32P66 | CXXC motif containing zinc binding protein | CZIB |
| A5PK65 | D-dopachrome decarboxylase | DDT |
| Q2KIT4 | DnaJ homolog subfamily B member 4 | DNAJB4 |
| A0A3Q1M2V8 | Dedicator of cytokinesis 1 | DOCK1 |
| Q32LC3 | Eukaryotic translation initiation factor 1A X-linked | EIF1AX |
| E1BLZ8 | Eukaryotic translation initiation factor 3 subunit F | EIF3F |
| Q3ZCK1 | Eukaryotic translation initiation factor 3 subunit L | EIF3L |
| G3N094 | EARP complex and GARP complex interacting protein 1 | EIPR1 |
| Q645M6 | FAS-associated death domain protein | FADD |
| Q2KIB0 | Fumarylacetoacetate hydrolase domain-containing protein 2 | FAHD2 |
| A7MBD4 | FARSA protein | FARSA |
| A0A3Q1LMA3 | Filamin A | FLNA |
| Q2KIM0 | Tissue alpha-L-fucosidase | FUCA1 |
| Q9MYM4 | Lysosomal alpha-glucosidase | GAA |
| Q5E9X0 | GDNF family receptor alpha-2 | GFRA2 |
| P15103 | Glutamine synthetase | GLUL |
| P38409 | Guanine nucleotide-binding protein subunit alpha-11 | GNA11 |
| A7MBH9 | G protein subunit alpha i2 | GNAI2 |
| F1MHQ1 | Glycoprotein A33 | GPA33 |
| A0A3Q1LLM4 | Histone deacetylase 10 | HDAC10 |
| P30546 | Histamine H1 receptor | HRH1 |
| F6S1Q0 | Keratin 18 | KRT18 |
| Q05204 | Lysosome-associated membrane glycoprotein 1 | LAMP1 |
| P02754 | Beta-lactoglobulin | LGB |
| A0A3Q1M7Z2 | Uncharacterized protein | LOC112441502 |
| A0A3Q1LQR2 | Tubulin alpha chain | LOC112443216 |
| F6PRC3 | Uncharacterized protein | LOC508153 |

**Supplementary Table 1. (continued)**

| **UniPort accession no.** | **Protein name** | **Gene name** |
| --- | --- | --- |
| E1BJE3 | Uncharacterized protein | LOC516378 |
| A0A3Q1LKP8 | Lipin 1 | LPIN1 |
| F6R1D7 | Family with sequence similarity 84 member B | LRATD2 |
| A0A3S5ZP87 | Leucine rich repeat containing 47 | LRRC47 |
| Q29451 | Lysosomal alpha-mannosidase | MAN2B1 |
| Q148C8 | Migration and invasion enhancer 1 | MIEN1 |
| Q5I598 | Methylenetetrahydrofolate reductase | MTHFR |
| Q9BE40 | Myosin-1 | MYH1 |
| Q9BE41 | Myosin-2 | MYH2 |
| A0A3Q1LN81 | N-acetyl-alpha-glucosaminidase | NAGLU |
| Q3T0Q4 | Nucleoside diphosphate kinase B | NME2 |
| F1MDI7 | P2Y purinoceptor 2 | P2RY2 |
| A5D7F4 | PCDHGC3 protein | PCDHGC3 |
| Q2TBR6 | Prefoldin subunit 4 | PFDN4 |
| Q0IIG5 | ATP-dependent 6-phosphofructokinase, muscle type | PFKM |
| A5PKH8 | PGM2L1 protein | PGM2L1 |
| F6QJJ8 | Progesterone receptor membrane component 2 | PGRMC2 |
| F1N7M1 | Phosphatidylinositol-4-phosphate 5-kinase type 1 beta | PIP5K1B |
| A0A3Q1LXA8 | Pyrophosphatase (inorganic) 2 | PPA2 |
| A0A3Q1N164 | PTPRF interacting protein alpha 1 | PPFIA1 |
| F1MMZ0 | Protein phosphatase 1 regulatory subunit 16A | PPP1R16A |
| F1MTZ0 | Protein phosphatase inhibitor 2 | PPP1R2 |
| Q1LZE9 | Serine protease 23 | PRSS23 |
| A5PK69 | RAC3 protein | RAC3 |
| P62833 | Ras-related protein Rap-1A | RAP1A |
| A0A3Q1LNY5 | RELA proto-oncogene, NF-kB subunit | RELA |
| Q2KIS1 | N-acylglucosamine 2-epimerase | RENBP |
| Q1RMJ6 | Rho-related GTP-binding protein RhoC | RHOC |
| F1MK42 | Ras homolog family member Q | RHOQ |
| Q3T0U2 | 60S ribosomal protein L14 | RPL14 |

**Supplementary Table 1. (continued)**

| **UniPort accession no.** | **Protein name** | **Gene name** |
| --- | --- | --- |
| F1ML72 | Ribosomal protein L34 | RPL34 |
| Q2TBQ5 | 60S ribosomal protein L7a | RPL7A |
| A0A3Q1LYD5 | Secretory carrier-associated membrane protein | SCAMP1 |
| E1BG76 | Sec1 family domain containing 1 | SCFD1 |
| A2I7M9 | Serpin A3-2 | SERPINA3-2 |
| A5PJK0 | Serpin B10 | SERPINB10 |
| E1BIF7 | SH3 domain binding protein 5 | SH3BP5 |
| A0A3Q1MPH1 | Solute carrier family 31 member 1 | SLC31A1 |
| P48765 | Sodium/calcium exchanger 1 | SLC8A1 |
| A0A3Q1LVA8 | Sorting nexin 12 | SNX12 |
| A0A3Q1LWU2 | Spectrin beta chain | SPTBN2 |
| F1MI92 | Slingshot protein phosphatase 3 | SSH3 |
| A0A3Q1NDL5 | Uncharacterized protein | SVIP |
| F1MIX5 | Tachykinin receptor 1 | TACR1 |
| A0A3Q1MFR6 | Uncharacterized protein | TMED2 |
| Q3ZC25 | Transmembrane protein 106B | TMEM106B |
| Q5KR47 | Tropomyosin alpha-3 chain | TPM3 |
| Q3ZCC8 | Tubulin polymerization-promoting protein family member 3 | TPPP3 |
| A6QLC7 | Trafficking protein particle complex subunit 11 | TRAPPC11 |
| A0A3Q1M442 | Tubulin beta chain | TUBB1 |
| Q0V7N0 | Vesicle-associated membrane protein 1 | VAMP1 |
| A2VDZ9 | Vesicle-associated membrane protein-associated protein B | VAPB |
| A0A3Q1M182 | Vav guanine nucleotide exchange factor 2 | VAV2 |
| A0A3Q1M7X4 | WD repeat domain, phosphoinositide interacting 1 | WIPI1 |
| E1BCF8 | Zinc finger CCCH-type containing, antiviral 1 | ZC3HAV1 |
| E1BK60 | Palmitoyltransferase | ZDHHC14 |
| Q58DA8 | Palmitoyltransferase ZDHHC9 | ZDHHC9 |
| G3MZ19 | Uncharacterized protein | ZG16B |
